# Fatal pediatric paraquat poisoning following scalp application for head lice treatment: Public health implications for pesticide regulation and emergency care — A case series

**DOI:** 10.1016/j.toxrep.2026.102272

**Published:** 2026-05-08

**Authors:** Amos Solomon, Paul Adah Omachi, Edith Chinwe Eze, Grace Ahmed, Olugoke Ezekiel Ojewole, Ayotunde Eniola Oyeleye, Usman Abdullahi, Abubakar Mammud, Hadiza Abdullahi, Zahra Fatima Idris, Ummukulthum Idris, Usman Abubakar

**Affiliations:** a Department of Paediatrics, Federal Medical Centre, Bida, Niger State, Nigeria; b Department of Family Medicine, Federal Medical Centre, Bida, Niger State, Nigeria

**Keywords:** Paraquat poisoning, Pediatric poisoning, Dermal exposure, Head lice treatment, Public health regulation, Low- and middle-income countries

## Abstract

Pesticide-related poisoning remains an important but preventable public health problem in children, particularly in settings with limited regulatory control. Paraquat is a highly toxic herbicide associated with severe systemic organ damage and high mortality following human exposure. Although ingestion is the most common route of poisoning, dermal absorption can result in fatal toxicity, especially with prolonged contact or compromised skin integrity. We report a rare case series of three female siblings aged 4, 6, and 8 years who developed severe paraquat toxicity following scalp application of the chemical as a home remedy for head lice. In this setting, scalp shaving is commonly practiced as part of lice treatment, often resulting in abrasions that may enhance dermal absorption. All three children presented approximately 36 h after exposure with features of systemic toxicity. The youngest child, who had more extensive scalp injury, rapidly developed respiratory distress and died shortly after presentation despite emergency resuscitation. The two older siblings initially remained conscious but subsequently developed progressive acute kidney injury and worsening respiratory failure requiring intensive care admission, haemodialysis, corticosteroid therapy, oxygen supplementation, and supportive management. Laboratory investigations demonstrated rising urea and creatinine levels with transient improvement following dialysis before subsequent deterioration. Despite aggressive supportive care, the 6-year-old child died on the ninth day of admission, while the 8-year-old died on the tenth day due to progressive respiratory failure and multi-organ dysfunction. The variation in clinical progression among the siblings likely reflects differences in the extent of scalp injury, degree of dermal absorption, exposure dose, and individual susceptibility. This case series highlights the extreme toxicity of paraquat, the absence of a specific antidote, and the devastating consequences of inappropriate domestic use of agricultural chemicals. It further emphasizes the urgent need for improved public awareness, stricter regulatory enforcement, and strengthened preventive strategies to reduce pediatric exposure and associated mortality.

## Highlights

• Accidental scalp paraquat use caused fatal pediatric poisoning.
• Dermal uptake led to AKI and progressive respiratory failure.
• All died despite dialysis, steroids, and ICU care.
• No antidote; paraquat has very high fatality rates.
• Stronger regulation and public awareness are urgently needed.

## Graphical Abstract

**Figure:**
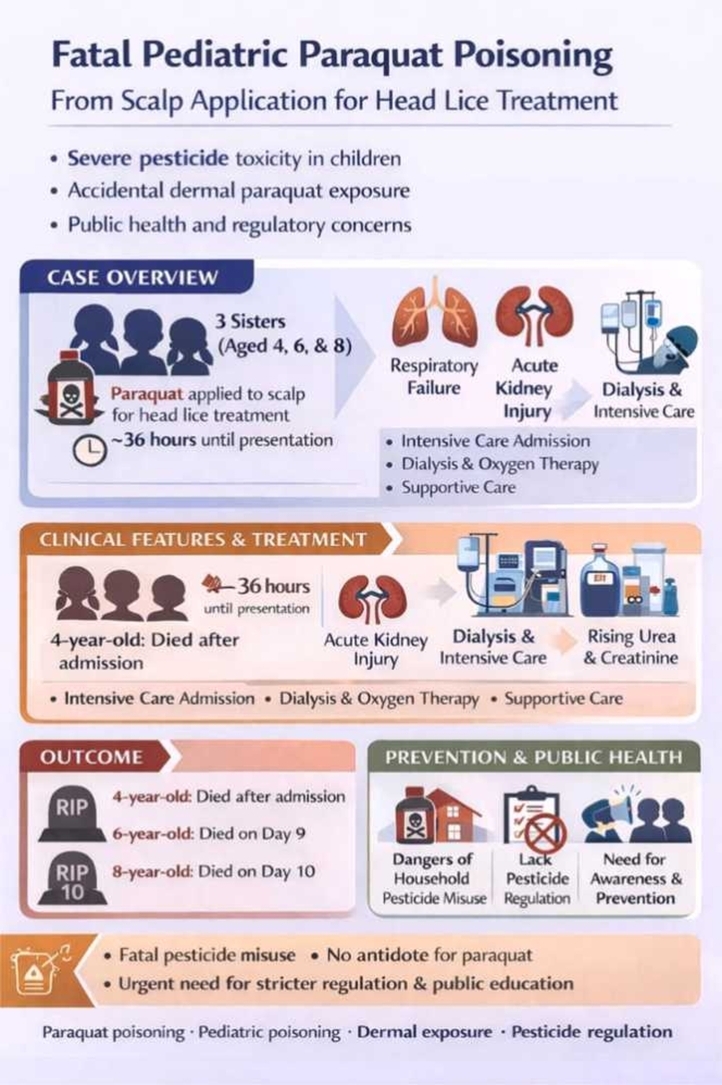

## 1 Introduction

Paraquat is a potent, non-selective herbicide that has been widely used in agriculture since its commercial introduction in the early 1960s. Chemically known as paraquat dichloride or methyl viologen, it belongs to the bipyridyl family of compounds and remains popular because of its rapid contact action and effectiveness against weeds and grasses [1].

Despite its agricultural utility, paraquat is extremely toxic to humans and animals even at small doses. Its toxicity is mediated through the generation of reactive oxygen species that induce oxidative stress, lipid peroxidation, and widespread cellular injury [2], [3], [4]. After systemic exposure, paraquat preferentially accumulates in the lungs and kidneys, resulting in progressive pulmonary inflammation, fibrosis, and acute tubular necrosis, which together account for the majority of fatal outcomes [2], [4].

Human exposure occurs primarily through ingestion, inhalation, ocular contact, and dermal absorption. Ingestion remains the most commonly reported and most lethal route; however, dermal exposure may also result in severe systemic toxicity, particularly when there is prolonged contact or disruption of the skin barrier [5], [6], [7], [8], [9]. This risk is significantly increased when exposure involves damaged skin surfaces, which may facilitate rapid systemic absorption [10].

Paraquat poisoning carries a high case-fatality rate, often exceeding 60–70% in severe exposures despite aggressive supportive care [4], [11], [12]. Global epidemiological data indicate that paraquat is responsible for a substantial proportion of pesticide-related deaths worldwide, particularly in low- and middle-income countries where regulatory oversight is limited [13], [14].

A major challenge in paraquat poisoning is the absence of a proven specific antidote. Current management strategies rely largely on supportive care, early decontamination, enhanced elimination techniques such as haemoperfusion or haemodialysis, and immunomodulatory therapies aimed at limiting pulmonary injury [14], [15], [16], [17]. The lack of targeted antidotal therapy contributes significantly to the poor prognosis associated with severe poisoning [17].

Regulatory responses to paraquat vary globally. Several countries, including members of the European Union and China, have banned or restricted its use due to public health concerns [18]. Evidence suggests that pesticide regulation is associated with reductions in poisoning incidence and mortality [8], [18], [19], [20]. However, in many developing countries, paraquat remains readily available through informal agricultural supply chains, often sold without prescription or adequate user education, increasing the risk of misuse and accidental exposure [7], [18], [21].

Reports of non-agricultural misuse and unusual exposure routes, including dermal absorption, have been documented and are associated with severe clinical outcomes [6], [9], [10], [22]. In some settings, paraquat and similar agricultural chemicals may be inappropriately used for domestic purposes, including topical application for conditions such as head lice, often following scalp shaving practices that can result in abrasions and compromise the skin barrier. These practices create a potential pathway for enhanced dermal absorption and systemic toxicity, particularly in children [10], [22].

These observations underscore the urgent need for improved regulatory enforcement and community education to prevent avoidable poisonings, particularly among vulnerable pediatric populations.

## 2 Case presentation

### 2.1 Case 1: 4-year-old girl

The youngest sibling was exposed to paraquat following scalp application after complete shaving of the hair as part of a home remedy for head lice. The chemical was obtained locally and applied directly onto the freshly shaved scalp. The chemical was not washed off the scalp and no immediate medical care was sought before presentation. Approximately 36 h later, the child developed sudden onset severe respiratory distress, and was brought to the emergency department. On arrival, she was conscious but in marked respiratory distress with hypoxemia. Physical examination revealed multiple scalp abrasions, excoriations and ulcerative lesions. Her condition deteriorated rapidly within minutes, progressing to cardiorespiratory collapse. Despite immediate resuscitative efforts, including airway support and advanced life support measures, she suffered cardiac arrest and died shortly after presentation. Laboratory investigations were not performed due to the rapid progression.

### 2.2 Case 2: 6-year-old girl

The second sibling presented at the same time with mild respiratory symptoms and initially stable vital signs. She was conscious and oriented at presentation, with complaints of mild cough, throat irritation, and early respiratory discomfort.

Scalp examination showed complete hair shaving with multiple superficial abrasions, excoriated areas, and shallow ulcerative lesions, some with mild serous discharge, similar to findings observed in her younger sibling.

**Fig. 1:**
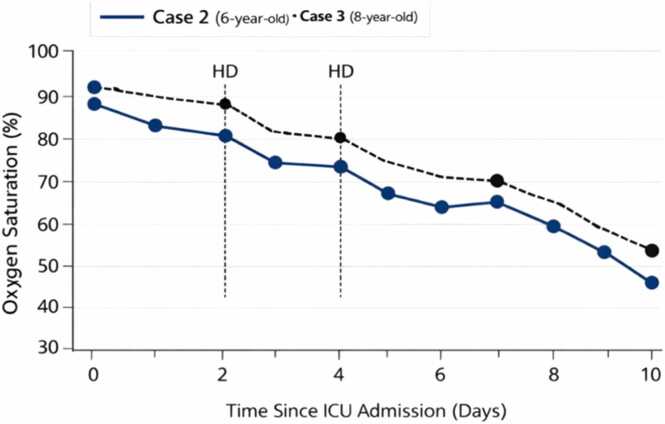
Trend in oxygen saturation in cases 2 and 3. This figure illustrates the progressive decline in oxygen saturation levels in both patients despite judicious oxygen therapy, reflecting worsening respiratory compromise.

**Fig. 2:**
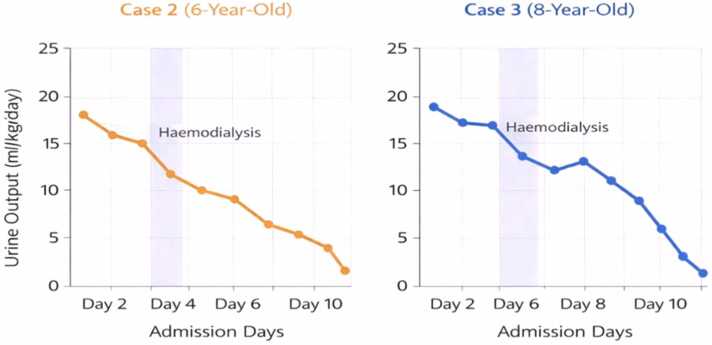
Trend in renal function parameters in cases 2 and 3. Serial measurements of serum creatinine and urea demonstrate progressive renal impairment, with transient improvement following haemodialysis and subsequent deterioration.

Within the first 24 h of hospitalization, she developed oliguria with rising serum creatinine, consistent with acute kidney injury. This was followed by worsening tachypnoea and declining oxygen saturation, necessitating admission to the intensive care unit (ICU). She received haemodialysis, corticosteroids, oxygen therapy, and supportive care. Although there was transient biochemical improvement following dialysis, her condition deteriorated progressively leading to severe respiratory failure and subsequent death on the ninth day of hospitalization (Table 1, Table 2; Fig. 1, Fig. 2).

**Table 1:** Serial laboratory parameters in case 2 (6-year-old girl).

**Day**	**Creatinine** **(µmol/L)**	**Urea** **(mmol/L)**	**Potassium** **(mmol/L)**	**Sodium** **(mmol/L)**	**Chloride** **(mmol/L)**	**Bicarbonate** **(mmol/L)**
1	290	11.6	4.9	134	102	18
2	350	14.8	5.7	132	104	16
3	300	9.5	4.4	136	100	21
3 (post-HD)	230	4.5	3.4	140	102	22
4	370	12.2	5.6	133	103	17
5	400	13.8	6.1	131	105	15
6	345	10.4	4.9	135	101	20
6 (post-HD)	156	4.2	3.2	138	106	22
7	410	14.2	4.9	134	108	12
8	450	16.7	5.7	137	108	10

HD = haemodialysis. Reference ranges: Creatinine (35–90 µmol/L), Urea (2.5–6.5 mmol/L), Potassium (3.5–5.0 mmol/L), Sodium (135 – 145 mmo/L), Chloride (98 −107 mmol/L), Bicarbonate (22 −28 mmol/L)

**Table 2:** Serial laboratory parameters in case 3 (8-year-old).

**Day**	**Creatinine** **(µmol/L)**	**Urea** **(mmol/L)**	**Potassium** **(mmol/L)**	**Sodium** **(mmol/L)**	**Chloride** **(mmol/L)**	**Bicarbonate** **(mmol/L)**
1	325	12.3	4.8	135	101	19
2	375	15.5	5.5	133	104	17
3	475	28.2	5.7	137	99	15
3 (post-HD)	345	13.2	5.0	135	100	22
4	365	14.9	5.6	134	99	18
5	400	18.4	4.9	130	99	18
6	400	18.6	4.8	130	99	14
6 (post-HD)	345	14.2	3.5	135	100	21
7	430	18.5	6.4	129	97	13
8	450	20.5	7.1	125	85	9

HD = haemodialysis. Reference ranges: Creatinine (35–90 µmol/L), Urea (2.5–6.5 mmol/L), Potassium (3.5–5.0 mmol/L), Sodium (135 – 145 mmo/L), Chloride (98 −107 mmol/L), Bicarbonate (22 −28 mmol/L)

### 2.3 Case 3: 8-year-old girl

The eldest sibling also presented at the same time after paraquat exposure via scalp application. At admission, she was fully conscious and initially appeared clinically stable, although she reported mild respiratory symptoms similar to her siblings.

Examination of the scalp revealed recent shaving with widespread excoriations, superficial abrasions, and small ulcerated areas, consistent with scalp trauma and repeated scratching. Some lesions showed mild serous exudation, suggesting disruption of the epidermal barrier.

Over the subsequent days, she developed acute kidney injury, evidenced by rising serum creatinine levels and reduced urine output, necessitating admission to the ICU and initiation of haemodialysis. Her respiratory status progressively worsened, with increasing oxygen requirements and persistent hypoxemia. Despite aggressive supportive therapy, including corticosteroids and ventilatory support, her condition continued to deteriorate (Table 1, Table 2; Fig. 1, Fig. 2). She subsequently developed multi-organ failure dominated by severe respiratory compromise and succumbed to the effects of paraquat toxicity on the tenth day of hospitalization.

### 2.4 Investigations and Laboratory Findings

Laboratory evaluation was not performed for the youngest sibling due to rapid clinical deterioration. In contrast, both older siblings demonstrated evidence of renal impairment at presentation. For the 6-year-old, admission values showed markedly elevated serum creatinine (290 µmol/L, (reference range 35–90 µmol/L)) and urea (11.6 mmol/L (reference range 2.5–6.5 mmol/L)) with mild electrolyte imbalance and metabolic acidosis. Similarly, the 8-year-old had elevated serum creatinine (325 µmol/L) and urea (12.5 mmol/L), with comparable electrolyte findings (Table 1, Table 2). Baseline oxygen saturation in both patients ranged from 88% to 92% on room air (Fig. 1), indicating early respiratory compromise.

Over the subsequent 24–48 h, both children exhibited progressive worsening of renal function (Table 1, Table 2), characterized by rising urea and creatinine levels and declining urine output, progressing from oliguria to near-anuria (Fig. 2). Although haemodialysis resulted in transient biochemical improvement, this was followed by rebound deterioration, reflecting ongoing systemic toxicity. Notably, liver function parameters remained largely within normal limits, suggesting minimal hepatic involvement. Concurrently, progressive respiratory compromise was observed, evidenced by declining oxygen saturation (Fig. 1), increasing oxygen requirements, and eventual need for ventilatory support.

Overall, the clinical and laboratory trends were consistent with evolving multi-organ dysfunction, predominantly affecting the renal and respiratory systems, with a transient response to therapy followed by progressive deterioration.

### 2.5 Basis for the diagnosis of paraquat poisoning

In the absence of toxicological confirmation, the diagnosis of paraquat poisoning was established based on a detailed exposure history, verification of the implicated substance, and a characteristic clinical course. Caregivers reported that the children’s scalps were shaved as part of a home remedy for head lice, after which a locally purchased chemical was applied topically. The substance, obtained from an agricultural supply outlet and dispensed into a tied nylon sachet, was identified by the caregivers as “Gamaline 20,” a paraquat-containing herbicide. To further ascertain the specific agent and guide management, caregivers were requested to retrieve the original container from the point of purchase, which confirmed paraquat as the active ingredient. Clinical examination revealed multiple scalp abrasions and areas of excoriation with serous discharge, consistent with mechanical injury from shaving and scratching, and indicative of a compromised skin barrier that would facilitate dermal absorption. The subsequent clinical progression characterized by early acute kidney injury, progressive hypoxaemic respiratory failure, transient biochemical improvement following haemodialysis, and eventual fatal outcomes was consistent with systemic paraquat toxicity as described in the literature [4], [6], [7], [10].

### 2.6 Management and outcome

There is currently no specific antidote for paraquat poisoning, and management remains largely supportive, aimed at limiting further absorption, enhancing elimination, and mitigating end-organ damage [15], [16]. Upon admission, the two older siblings were commenced on aggressive supportive therapy, including close monitoring of vital parameters, cautious oxygen supplementation to avoid risk of oxygen-enhanced oxidative injury, and correction of fluid and electrolyte abnormalities.

Both patients developed progressive acute kidney injury with oliguria and rising serum creatinine levels, necessitating initiation of haemodialysis. Each child underwent two sessions of haemodialysis (on days 3 and 6 of admission), which resulted in transient biochemical improvement, including partial reduction in serum urea and creatinine levels (Table 1, Table 2; Fig. 1, Fig. 2). However, renal function subsequently deteriorated, reflecting ongoing systemic toxicity and continued tissue injury.

Systemic corticosteroid therapy was instituted using intravenous hydrocortisone, in the absence of methylprednisolone, with the aim of attenuating pulmonary inflammation and limiting progression to fibrosis. This approach is supported by previous studies that have explored immunomodulatory therapy in paraquat-induced lung injury, although outcomes remain inconsistent [14], [17], [23], [24].

Maintenance intravenous fluids were administered using 5% dextrose-based solutions, with careful titration to avoid fluid overload while maintaining adequate perfusion. Electrolyte imbalances were corrected as required. Both patients required escalating oxygen therapy due to worsening hypoxemia and progressive respiratory compromise. Despite intensive care support, including ventilatory assistance in the later stages, both children experienced relentless deterioration of pulmonary function.

Although all three siblings were exposed at approximately the same time, differences were observed in the timing and progression of clinical deterioration. These variations likely reflect differences in the extent of dermal exposure, degree of scalp injury, body surface area (BSA) and individual physiological susceptibility, which may have influenced the rate of systemic absorption and subsequent organ injury.

The 6-year-old child died on the ninth day of admission from progressive respiratory failure, while the 8-year-old child succumbed on the tenth day following multi-organ failure. The youngest sibling died shortly after arrival due to fulminant respiratory collapse. These outcomes reflect the well-documented high fatality rate associated with paraquat poisoning, even in settings with access to dialysis and critical care support [4], [12].

## 3 Discussion

Paraquat poisoning remains one of the most severe forms of herbicide toxicity encountered in clinical practice worldwide. Its mechanism of injury is driven by free radical generation and oxidative stress, leading to extensive cellular damage [2], [3]. The lungs and kidneys are particularly vulnerable due to selective uptake of paraquat into alveolar epithelial cells and renal tubular cells, resulting in progressive pulmonary fibrosis and acute kidney injury characterized by rising urea and creatinine levels, oliguria, and electrolyte disturbances [2], [3], [4]. These pathological processes often culminate in multi-organ failure and high mortality. The clinical course observed in the older siblings in this case series mirrors these established pathophysiological patterns.

Mortality associated with paraquat poisoning remains uniformly high, particularly in cases of delayed presentation or significant systemic absorption. Large clinical series have consistently reported fatality rates exceeding 60%, even in settings with access to intensive care support [11], [12]. The unusual route of exposure in the present cases (dermal absorption through scalp application) is rare but clinically significant. Previous reports have demonstrated that paraquat can be systemically absorbed through damaged skin, leading to severe toxicity comparable to that seen with oral ingestion [7], [9]. Dermal paraquat toxicity is well described; however, exposure via the scalp is extremely rare. To our knowledge, only one prior report has documented fatal poisoning following skin absorption [10], with no similar pediatric cases identified. The present report therefore represents a unique contribution, not only because of the scalp route of exposure but also due to the occurrence of three affected siblings from a single exposure event.

An important contextual factor in this case series is the local practice of managing pediculosis (head lice infestation). In many low-resource settings, complete shaving of the scalp is commonly performed to reduce lice burden. This is often followed by application of topical substances frequently without medical guidance with the belief that these will eliminate residual lice and nits. In the present cases, caregivers reported applying a paraquat-containing product after shaving, likely reflecting a lack of awareness of its toxicity and inappropriate repurposing of an agricultural chemical for domestic use. This practice explains why the chemical was applied despite shaving, and highlights a critical gap in community knowledge and pesticide safety education.

The presence of multiple scalp abrasions, excoriations, and ulcerative lesions observed in all three children is also of clinical significance. These lesions were consistent with mechanical trauma sustained during shaving with razor blades and subsequent scratching, rather than pre-existing dermatological disease. The disruption of the epidermal barrier created an ideal pathway for enhanced dermal absorption of paraquat. It is well established that intact skin offers some protection against toxin absorption; however, compromised skin integrity significantly increases systemic uptake, thereby accelerating toxicity [9], [10].

A notable finding in this case series is the variation in the onset and progression of clinical deterioration among the three siblings, despite a similar exposure timeline. The youngest child developed rapid respiratory compromise and died shortly after presentation, whereas the older siblings exhibited a more protracted course, with death occurring on days 9 and 10 of hospitalization. This variation can be plausibly explained by differences in the extent of scalp injury, degree of dermal absorption, quantity of chemical exposure, and individual biological susceptibility, including age-related physiological differences. The youngest child, who demonstrated more extensive scalp compromise, likely experienced more rapid systemic absorption, leading to fulminant toxicity. In contrast, the older siblings may have had relatively lower absorption rates, resulting in a delayed but ultimately fatal progression. These observations underscore the heterogeneity of clinical manifestations even within the same exposure context.

Within Nigeria, paraquat poisoning has been reported in the literature, although documented cases remain relatively few. A case from Ondo State described acute toxic nephropathy following paraquat ingestion, highlighting the burden of renal complications associated with this toxin in local clinical practice [2], [5]. More recently, a case report from Delta State documented severe paraquat poisoning in a pregnant adolescent, with rapid progression to multi-organ failure despite hospital intervention [1]. These reports demonstrate that paraquat toxicity represents a real and ongoing clinical threat within the Nigerian healthcare system. However, reports involving dermal exposure particularly in children are exceedingly rare, further emphasizing the uniqueness of the present series.

Management of paraquat poisoning remains particularly challenging. Unlike organophosphate poisoning, for which specific antidotes exist, paraquat lacks a targeted reversal agent [13]. Haemodialysis and haemoperfusion may reduce circulating toxin levels and correct metabolic derangements, but they do not prevent ongoing pulmonary uptake or halt fibrotic progression [6], [15], [16], [22]. Immunosuppressive regimens using corticosteroids have been employed in attempts to attenuate inflammatory lung injury, though clinical outcomes remain inconsistent [8], [14], [17]. In resource-limited settings such as Nigeria, delayed access to dialysis, limited ICU capacity, and absence of specialized toxicology services further complicate management and worsen prognosis. These cases further underscore the need for strengthened emergency preparedness, including early triage protocols, access to renal replacement therapy, and clinician training in the recognition of non-oral pesticide exposures [1], [2], [5], [7], [10].

The clinical trajectory observed in this case series characterized by initial stabilization followed by progressive respiratory and renal deterioration, reflects patterns described in high-fatality international cohorts [7], [9], [10]. Once significant systemic absorption has occurred, supportive care alone is often insufficient to reverse disease progression.

On the regulatory front, Nigeria has recently taken steps toward restricting paraquat use following growing evidence of its public health impact [23], [25]. However, enforcement remains a major challenge. Informal markets and weak surveillance mechanisms allow continued circulation of paraquat products, particularly in rural communities [25]. Without sustained regulatory monitoring, public education, and safe disposal of existing stockpiles, accidental and inappropriate domestic use is likely to persist, as illustrated by this case series.

These findings highlight the urgent need for clinician awareness, early recognition of atypical exposure routes, and improved preventive strategies. While timely supportive care remains essential, meaningful reduction in paraquat-related morbidity and mortality will depend on strengthened regulatory enforcement, safer pesticide distribution practices, and community-level education to prevent hazardous domestic misuse.

## 4 Conclusion

Accidental paraquat exposure via scalp application is a rare but highly lethal form of poisoning in children, as seen in this case series involving inappropriate use of an agricultural chemical after routine head shaving for lice treatment. Scalp abrasions from shaving likely enhanced dermal absorption. The varying clinical courses, from rapid deterioration in the youngest child to a more gradual decline in older siblings, reflect differences in exposure extent, skin integrity, and individual susceptibility. Despite intensive supportive care, all three children died, underscoring the extreme toxicity of paraquat and the lack of an effective antidote. These highlights significant gaps in public awareness, unsafe pesticide practices, and weak regulatory control in low-resource settings. Prevention remains critical, requiring improved community education, stricter regulation of hazardous chemicals, and heightened clinical suspicion of atypical exposure routes.

## 5 Recommendations

There is an urgent need for intensified public awareness campaigns targeting caregivers, farmers, and agrochemical vendors on the dangers of herbicide misuse and the risks associated with domestic application of agricultural chemicals. Regulatory authorities should strengthen enforcement of Nigeria’s paraquat ban through enhanced market surveillance, restriction of informal sales, and safe disposal of existing stockpiles. In parallel, the development of national clinical guidelines for paraquat poisoning, establishment of clear early referral pathways, and improved access to renal replacement therapy and critical care services are essential to strengthen emergency response capacity and improve clinical outcomes following severe toxic exposures.

## 6 Limitations

This report is limited by the absence of quantitative serum paraquat measurements and confirmatory toxicological testing, which restricted objective assessment of toxin burden. Specifically, the unavailability of bedside urine sodium dithionite testing and blood paraquat level estimation limited the ability to confirm exposure and quantify severity. Histopathological evaluation was also not performed. However, the diagnosis was supported by a clear exposure history, identification of the implicated chemical, and a consistent clinical course across all cases.

## CRediT authorship contribution statement

**Grace Ahmed:** Investigation. **Olugoke Ezekiel Ojewole:** Methodology, Investigation. **Ayotunde Eniola Oyeleye:** Investigation. **Usman Abdullahi:** Validation, Investigation. **Ummukulthum Idris:** Investigation. **Amos Solomon:** Writing – original draft, Data curation, Conceptualization. **Usman Abubakar:** Supervision. **Paul Adah Omachi:** Writing – review & editing, Methodology. **Edith Chinwe Eze:** Methodology, Investigation. **Abubakar Mammud:** Investigation. **Hadiza Abdullahi:** Investigation. **Zahra Fatima Idris:** Investigation.

## Consent for Publication

Informed consent was obtained from the child’s caregivers.

## Ethics approval

Ethical approval was waived for this retrospective case series, as it involved anonymized routine clinical data and contained no direct patient identifiers.

## Funding

No funding was received for this study.

## Declaration of Competing Interest

The authors declare that they have no known competing financial interests or personal relationships that could have appeared to influence the work reported in this paper.

## Data Availability

Data will be made available on request.
